# Highlights of the Fourth Canadian Symposium on Hepatitis C: Moving towards a National Action Plan

**DOI:** 10.1155/2016/5743521

**Published:** 2016-04-27

**Authors:** Selena M. Sagan, Benoit Dupont, Jason Grebely, Mel Krajden, Sonya A. MacParland, Jennifer F. Raven, Sahar Saeed, Jordan J. Feld, D. Lorne Tyrrell, Joyce A. Wilson

**Affiliations:** ^1^Department of Microbiology & Immunology, McGill University, Montréal, QC, Canada H3A 2B4; ^2^Liver Unit, Department of Medicine, Université de Montréal, Montréal, QC, Canada H3T 1J4; ^3^The Kirby Institute, UNSW Australia, Sydney, NSW 2052, Australia; ^4^British Columbia Centre for Disease Control, Vancouver, BC, Canada V5Z 4R4; ^5^University of British Columbia, Vancouver, BC, Canada V6T 1Z4; ^6^Toronto General Research Institute, University Health Network, Toronto, ON, Canada M5G 1L7; ^7^Canadian Institutes of Health Research-Institute of Infection and Immunity, Ottawa, ON, Canada K1A 0W9; ^8^Department of Epidemiology, McGill University, Montréal, QC, Canada H3A 1A2; ^9^Toronto Western Hospital Liver Center, University Health Network, Toronto, ON, Canada M5T 2S8; ^10^Li Ka Shing Institute of Virology, Department of Medical Microbiology & Immunology, University of Alberta, Edmonton, AB, Canada T6G 2E1; ^11^Department of Microbiology & Immunology, University of Saskatchewan, Saskatoon, SK, Canada S7N 5E5

## Abstract

Hepatitis C virus (HCV) affects at least 268,000 Canadians and causes greater disease burden than any other infectious disease in the country. The Canadian Institutes of Health Research (CIHR) and the Public Health Agency of Canada (PHAC) have identified HCV-related liver disease as a priority. In 2015, the release of well-tolerated, short course treatments (~12 weeks) able to cure the majority of treated HCV patients revolutionized HCV therapy. However, treatment is extremely costly and puts a significant burden on the Canadian healthcare system. Thus, managing treatment costs and improving treatment engagement in those most in need will be a key challenge. Diagnosis and treatment uptake are currently poor in Canada due to financial, geographical, cultural, and social barriers. The United States, Australia, and Scotland all have National Action Plans to prevent, diagnose, and treat HCV in order to efficiently reduce the burden and costs associated with HCV-related liver disease. The theme of the 4th annual symposium held on Feb 27, 2015, “Strategies to Manage HCV Infection in Canada: Moving towards a National Action Plan,” was aimed at identifying strategies to maximize the impact of highly effective therapies to reduce HCV disease burden and ultimately eliminate HCV in Canada.

## 1. Introduction

With the release of interferon- (IFN-) free HCV therapies, research has attained the ultimate goal of developing a cure for HCV infection. New treatment combinations are highly effective [achieving sustained virological responses (SVR) in over 90% of people in clinical trials] and are well tolerated [1]. The advent of these new therapies represents a revolution in the ability to treat HCV-infected individuals and has been met with great excitement and optimism by the affected population and the physicians who treat them. However, given the large population of Canadians infected, many of whom are marginalized, a plan to identify those infected and engage them in care and treatment will be necessary. Without significant resources to increase treatment uptake, the goal of HCV elimination in Canada will remain elusive.

## 2. The NCRTP-HepC

The National CIHR Research training program in hepatitis C (NCRTP-HepC) is a Canadian Institutes of Health Research- (CIHR-) supported Strategic Training Initiative in Health Research established in 2003 (http://www.ncrtp-hepc.ca/). The NCRTP-HepC was supported by public funds from a partnership between CIHR and the Public Health Agency of Canada (PHAC) as well as by nongovernmental (e.g., the Canadian Liver Foundation), industry, as well as private and community organizations. The NCRTP-HepC was designed to foster translational research capacity, cross-disciplinary learning, and collaboration among clinical, basic biomedical, social, population health, and health systems/services researchers from fields including medicine, nursing, and social sciences. The overall goal of the program is to increase interdisciplinary Canadian research and training capacity and ultimately eliminate HCV disease in Canada within the next 10 to 15 years. The program consists of 36 leading researchers and clinicians from universities across Canada, who act as mentors for the trainees involved in Canadian HCV research. Since 2003, the NCRTP-HepC has supported 77 trainees (11 M.S., 39 Ph.D., 3 M.D., and 24 postdoctoral) and 53 summer students. This program has significantly enhanced HCV research capacity, knowledge translation/exchange, and interdisciplinary collaboration in Canada.

## 3. The 4th Canadian Symposium on HCV (CSHCV)

Over the past 4 years, the NCRTP-HepC has facilitated HCV research translation in Canada by organizing the CSHCV [2, 3]. In response to feedback from community groups and the first three symposia, the specific aims of the 4th CSHCV were as follows:

1. To discuss strategies to decrease HCV disease burden using the new highly effective therapies and build momentum for the development of a Canadian action plan.
2. To facilitate transdisciplinary knowledge exchange and collaborations between Canadian trainees, established researchers, healthcare practitioners, health policy makers, and community-based groups working on HCV.
3. To disseminate symposium findings to support practice change, community awareness, harm reduction, and treatment policy development.

A one-day symposium was held on Feb 27, 2015, only a few months after Health Canada's approval of highly effective IFN-free combination therapies for HCV infection. The theme of the meeting, “Moving towards a National Action Plan,” reflected the need for Canada to develop a rational plan outlining targets and key strategies to improve HCV prevention, management, and treatment, thereby reducing HCV-related disease burden. Some key questions included the following:

- How can effective prevention strategies be expanded to decrease the numbers of new cases of HCV infection?
- How can treatments be delivered and targeted to achieve the greatest impact?
- Reimbursement for HCV treatment is restricted to people with advanced liver disease; is this the best strategy given current recommendations and available data?
- Can the population-level impact of HCV treatment be improved by expanding access to those at risk of transmitting infection (e.g., people with HIV infection and people who inject drugs)?
- What strategies can be developed to engage marginalized populations (e.g., people who inject drugs, HIV coinfected, and Aboriginal people) into care?
- Will resistance to IFN-free therapy be a major clinical issue in the future?
- What is the incidence of HCV reinfection following successful IFN-free therapy among people with ongoing risk behaviours?
- How will the availability and demand for new IFN-free treatments alter HCV care in Canada?

Understanding how to use new therapies to provide better care for HCV-infected individuals will require integration between multiple fields of medical research, including biomedical and clinical sciences, health services, and social, cultural, environmental, and population health. The symposium brought together transdisciplinary research scientists, clinicians, nurses, community health workers, patient advocates, and public health officials to facilitate discussion of information needed to make informed decisions on priorities for HCV care in Canada. The title and authors of the presentations discussed in this symposium report are listed in Table 1.

### 3.1. Treatment for Chronic HCV Infection: Challenges and Opportunities in the Era of Highly Effective Antiviral Therapy

In the opening keynote presentation, Dr. Mark Sulkowski (Johns Hopkins University School of Medicine, Baltimore, USA) presented an overview of the basic research and clinical trials that led to the approval of currently available, highly effective combinations of IFN-free HCV therapies [4]. His presentation highlighted the rapid translation of laboratory findings to clinical therapy over the last 20 years and discussed the challenges and opportunities offered by new and effective HCV treatments.

The revolution in HCV therapy was made possible by the development of multiple direct-acting antivirals (DAAs) that target essential viral proteins, including the NS3 protease; NS5A, a protein required for virus replication and assembly; and NS5B, the viral polymerase. Combinations of DAAs of different classes have proven highly effective for most patients. With potent combination therapy, antiviral resistance has been less of a problem than originally anticipated, partially due to the very high barrier of resistance of the nucleotide polymerase inhibitor class of agents, including sofosbuvir, the first approved antiviral of this class [5, 6].

A brief history of HCV DAA development starts in 2003, with the first NS3 protease inhibitor, BILN2061 [7]. BILN2061 had very potent inhibitory activity but was discontinued during clinical trials due to cardiac toxicity in animal testing. This inhibitor was followed by telaprevir and boceprevir used in combination with IFN to treat genotype 1 HCV infections [8]. Subsequent advances included the development of the NS5A inhibitor, daclatasvir, which could induce a 4-log decline in HCV titres with a single dose, likely due to the potent inhibition of the multifunctional NS5A protein [9, 10]. However, these combination therapies still required the use of IFN and thus retained the associated side effects. In addition, many were genotype-specific which limited their usefulness. Importantly, viral resistance emerged quickly through selection of previously existing resistant mutants. Furthermore, these mutations frequently exhibited cross-resistance to other inhibitors with the same viral protein target.

As more antivirals became available, attempts were made to develop IFN-free combination therapies. The first cases of IFN-free cure were reported in 2012 using a combination of daclatasvir (NS5A) and asunaprevir (NS3); however, this combination was effective only in patients with genotype 1b, whereas those with genotype 1a quickly developed resistance [11–13]. These studies demonstrated that HCV could be eliminated from patients using a combination of DAAs, but that two inhibitors with a low barrier to resistance were not sufficient in most patients due to the rapid emergence of resistant variants. The addition of a nonnucleotide NS5B polymerase inhibitor, or the nucleoside analog ribavirin, in combination with NS3 and NS5A inhibitors, reduced but did not eliminate resistance development [14–17]. In addition to HCV subtype, it quickly became apparent that patients with cirrhosis were also difficult to cure, suggesting that intrahepatic immune cells found in cirrhotic livers may be dysfunctional or possibly that architectural changes in the cirrhotic liver affect drug distribution [15, 18]. Notably, coinfection with human immunodeficiency virus (HIV) did not strongly affect therapeutic efficacy with these new antivirals, a marked contrast to the poor results seen in the coinfected population with IFN-based therapies [19].

A key advance was the development of the nucleotide inhibitor sofosbuvir. This inhibitor targets the NS5B polymerase active site and has pan-genotypic activity. Importantly, because of the very poor replicative fitness of variants resistant to sofosbuvir, viral breakthrough is almost never seen during treatment with this drug and resistant variants rarely emerge, even in those who relapse [5, 6]. Sofosbuvir has been successfully used in combination with ribavirin, NS5A inhibitors (daclatasvir or ledipasvir), or an NS3 inhibitor (simeprevir), even in cirrhotic patients and those who had previously failed treatment [12, 17, 20–22]. Due to its high barrier to resistance, successful retreatment with sofosbuvir in combination with other antivirals may be possible even in patients who fail a sofosbuvir-based regimen [21]. Other antivirals of this class have met challenges with toxicity in early clinical trials. The results of several clinical trials have led to new guidelines recommending that HCV treatment should be based on oral drug combinations and should no longer include IFN (American Association for the Study of Liver Diseases (AASLD)/Infectious Disease Society of America (IDSA) HCV Guidelines, http://www.hcvguidelines.org/) [23]. The currently recommended combinations depend on simple criteria: virus genotype and subtype, the presence or absence of cirrhosis, and prior HCV treatment status.

In echo to Dr. Sulkowski's presentation, Dr. Emmanuelle Huchet (Clinique l'Actuel, Montréal, Quebec, Canada) presented an original study on the efficacy of sofosbuvir treatment regimens in real life settings [24]. Even though recent clinical trials demonstrated spectacular efficacy and tolerance of sofosbuvir-based treatments [21], there is limited data currently available on its real life use. Dr. Huchet's team conducted a prospective study on all genotype 1-infected patients treated with combination therapies that included sofosbuvir. Among 40 patients followed up long enough to assess SVR at 12 weeks, only 65% reached SVR. The factors associated independently with SVR were the absence of cirrhosis and the sofosbuvir/simeprevir combination therapy. These results highlight the fact that SVR rates are likely to be lower in populations consisting mostly of cirrhotic patients. These patients may also be in more difficult social situations than those observed in clinical trials that typically include highly selected populations. The therapeutic success rates outside of clinical trials remain to be established, but it is expected that within a few years highly tolerable, short-duration (6–12 weeks) therapy with extremely high efficacy (cure rates > 90%) will be the norm [25].

### 3.2. Biomedical Sciences: Mechanisms of Antiviral Inhibition and Viral Resistance

Related presentations highlight the contributions of basic scientists to the advances in drug discovery and virus resistance. The first speaker of this session, Dr. Volker Lohmann (University of Heidelberg, Heidelberg, Germany) [26] was responsible for one of the most important advances that allowed for the development of DAAs: the establishment of the replicon system to study HCV replication in cell culture [27]. In his presentation, Dr. Lohmann described his more recent efforts in understanding the mechanism(s) of action of NS5A inhibitors and focused on NS5A-induced membrane alterations, collectively known as the “membranous web” (MW) [26]. The MW is the site of HCV replication in hepatocytes and consists primarily of double-membrane vesicles (DMVs) that harbour viral replication complexes. NS5A is crucial for the formation of DMVs, and highly potent NS5A inhibitors, such as daclatasvir, decrease DMV size and number [28]. Experiments suggest that the inhibitors may “freeze” the NS5A protein in a particular dimer conformation and inhibit NS5A's association with essential host factors required for biogenesis of the MW and viral replication sites [29]. An NS5A interacting factor that is of particular interest to Dr. Lohmann is PI4KIII*α*. PI4KIII*α* is a lipid kinase that converts phosphatidylinositol to phosphatidylinositol 4-phosphate (PI4P) and has an important function in MW formation and HCV RNA replication [30]. Recent work suggests that it interacts directly with both NS5A and NS5B [30]. Together, these two viral proteins induce PI4KIII*α* to produce massive amounts of PI4P in HCV-infected cells. This results in recruitment of other lipid transporter proteins and thereby brings together all the components needed to create the MW and authentic HCV replication sites [31, 32]. Interestingly, tissue culture adaptive mutations in NS5A and NS5B in genotypes 1, 3, 4, and 5 abrogate activation of PI4K and enhance replication efficiency in cultured hepatoma cells [26]. Thus, evasion from unfavourably high PI4K expression levels in cell culture might be a limiting factor in the ability to culture wild-type HCV isolates* in vitro*. Understanding this mechanism might pave the way for culturing patient isolates in hepatoma cells, which is currently limited to a single viral isolate of genotype 2a.

In addition to understanding the mechanisms of action of DAAs, mechanisms of viral resistance to therapy are also an important and active area of research in multiple laboratories, including that of Dr. Matthias Götte (University of Alberta, Edmonton, Canada) [33]. Due to the rapid rate of replication and the error-prone nature of the viral NS5B RNA-dependent RNA polymerase, HCV exists as a population of genetically diverse but closely related viruses within each infected patient. Resistant variants are therefore already present before treatment initiation; however, most resistant variants replicate poorly and are undetectable prior to therapy. Once treatment is initiated, sensitive viruses are eliminated while resistant viruses survive and outgrow the population, resulting in rebound or viral breakthrough. Clinically relevant resistant variants identified* in vivo* exist for all classes of DAAs, including the NS3/4A protease inhibitors, NS5A inhibitors, and NS5B polymerase (nucleotide and nonnucleotide) inhibitors. For nucleotide inhibitors, like sofosbuvir, the main mutation that confers resistance is NS5B S282T. However, this mutation is rarely selected* in vitro* and* in vivo*. In recent clinical trials, >2000 patients were treated with sofosbuvir in mono- or combination therapy and only one subject exhibited evidence of sofosbuvir resistance [34]. In this subject, sofosbuvir monotherapy resulted in a rapid decline in viral load; however, during follow-up, there was a rebound of the virus and upon sequencing, the S282T mutation was identified. However, due to the low fitness of the S282T mutant, it was rapidly outgrown by wild-type virus and the subject was retreated with sofosbuvir/ribavirin to subsequently achieve a SVR [34, 35].

The two main issues that influence the rate at which resistant viruses emerge are (1) the genetic barrier and (2) viral fitness. The genetic barrier is the ease with which the polymerase can generate a specific resistance mutation. For example, transversion mutations (purine to pyrimidine or vice versa) are harder for the polymerase to accommodate than transition mutations (purine to purine or pyrimidine to pyrimidine) and hence have a higher genetic barrier to resistance [36]. Viral fitness refers to the capacity of a viral variant (i.e., one with a resistance-conferring mutation) to replicate in a given environment. This may help explain why the S282T mutation is relatively rare; it requires a transversion mutation that is not frequently generated, and it also has poor replicative fitness.

In a recent crystal structure of NS5B with a bound nucleotide and sofosbuvir, respectively, the S282 residue lies in close proximity to the polymerase active site where the substrate and inhibitor bind [37]. S282 and the adjacent G283 residue are part of a ring-like structure that coordinates the template RNA and the incoming (natural or analog) nucleotide during RNA synthesis. Dr. Götte presented data to support their hypothesis that the S282T mutation alters this structure and affects binding of sofosbuvir and, to a lesser extent, binding of the natural nucleotide substrate. This information provides an explanation for why the S282T mutant is resistant to sofosbuvir but also has reduced replicative fitness. Interestingly, susceptibility of ribavirin (a smaller nucleoside analogue inhibitor) is slightly increased in the context of S282T. This is yet another potential factor that helps to explain the rare selection of S282T in the context of sofosbuvir/ribavirin combination therapy. Together, these results shed light on the mechanism of resistance to sofosbuvir and provide a rationale for the combined use of sofosbuvir/ribavirin.

Efficient treatment using the well-tolerated DAA combinations offers numerous advantages to HCV-infected patients. Successful HCV treatment (SVR) is tantamount to a “virologic cure” and all patients are expected to benefit from this. At the population level, cure will reduce all-cause mortality and liver-related health adverse consequences, including end-stage liver disease and hepatocellular carcinoma [38]. Curative HCV therapies are the direct result of the remarkable progress in HCV research and the translation of laboratory and clinical discoveries into approved drugs, from discovery of HCV in 1989 to highly effective and tolerable treatments today. However, questions remain on “how low can you go” in treatment complexity and duration and on how to deal with drug resistance in persons who have viral breakthrough or relapse. Finally, future challenges remain in translating the clinical successes into global effectiveness in the population and this will require substantial public health initiatives.

### 3.3. Clinical Sciences: Treatment of HCV in Canada

Dr. Curtis Cooper (University of Ottawa, Ottawa, Ontario) provided a current portrait of HCV infection in Canada [39]. Among 268,000 HCV-infected patients in Canada, 58% are PWID (including 38% who are current intravenous drug users) and 20% have emigrated from high prevalence countries. The vast majority of infected patients are between 40 and 50 years old. However, a large number of HCV-infected Canadians remain undiagnosed. There is a current recommendation to systematically screen people born between 1945 and 1970 since it is predicted to identify 69% of undiagnosed HCV infections [40]. However, despite data supporting this recommendation, the PHAC has not yet formally advocated for birth cohort screening in Canada. The HCV-infected population is aging and subject to complications of chronic infection (cirrhosis, hepatocellular carcinoma, and liver-related death) with the incidence of these complications likely to peak in the next 20 years [41]. However, widespread use of the new therapies with the benefit of achieving SVR in the majority of patients could greatly reduce the occurrence of these complications [42]. Thus, the high cost of new treatments could be balanced by cost savings in the management of complications in untreated HCV infections [41]. Dr. Cooper emphasized the necessity of a coherent policy allowing the widest possible access to the new generations of treatment but also stressed that management of patients by specialized multidisciplinary teams is likely to still be required.

To further address the topic of which HCV patients should be treated first, Dr. Cooper and Dr. Jordan Feld (University Health Network, Toronto, Canada) engaged in a spirited debate entitled “Be it resolved that new HCV treatments should only be used on the sickest patients (F2 and above)” [43]. On the “pro” side, Dr. Cooper opened by stating that while he would provide the “pro” perspective of this topic, his debate points would not necessarily represent his personal beliefs. He suggested that, due to the cost of therapies, clinicians need to make strategic choices to treat the highest risk individuals first, namely, patients with advanced fibrosis. He stated that it is critical that we use proper patient follow-up to identify individuals with advanced fibrosis, which will allow the stratification of treatment based on need. While conceding that the ideal situation would be treatment for all HCV-infected patients at once, he suggested that due to the lack of government funding for treatment, coupled with the high costs of therapy, a well-organized approach to delivering the best possible care will need to be devised and presented to policy makers, in order to make a claim for increased funding for treatment delivery.

On the “con” side, Dr. Feld opened by stating that he was pleased to be presenting the “con” perspective and was happy in personal agreement with all the points he would be presenting. Dr. Feld mentioned nonliver HCV-related complications, unpredictable liver disease progression, the overall cost of HCV morbidity and mortality [41], as well as the goal of HCV elimination as the main reasons for treating everyone, regardless of their degree of fibrosis. He pointed out that the F2 rule originated from the era of IFN and was instituted due to the lack of efficacy coupled with the toxicity of IFN-based therapy, with the idea that only the people who had severe liver disease would be treated. He then proposed that these rules should no longer apply since IFN-free therapies are well-tolerated and result in health-related quality of life improvement during therapy [44] as well as a reduction in all cause-mortality after SVR [42]. He further stated that long-term follow-up for patients to determine when they will need therapy would be both challenging and expensive. In conclusion, Dr. Feld suggested that providing universal treatment will require creativity, including lowering the cost per pill, instituting a price per cure, or amortizing the cost of treatment over time.

In a continuation of the debate topic, Dr. Mel Krajden (University of British Columbia, BC, Canada) led a panel discussion [45]. The panel included Dr. Jordan Feld (University Health Network, Toronto, Canada); Ms. Magdalena Kuczynski (Toronto Western Hospital, Toronto, Canada); Dr. Patricia Bacon (Chair-Action Hepatitis Canada); Dr. Philippe Bourgois (University of Pennsylvania, Philadelphia, USA); Dr. Sharon Hutchinson (Glasgow Caledonian University, Glasgow, Scotland, UK); Dr. Lorne Tyrrell (University of Alberta, Edmonton, Canada); Dr. Julie Bruneau (Université de Montréal, Montreal, Canada); and Ms. Margaret Poitras (CEO, All Nations Hope Network, Regina, Canada). The overall perception was that Canada presently possesses all the ingredients needed for an effective HCV action plan, including research data, patient advocacy groups, and clinical champions. However, it was suggested that the difference between Canada and Scotland, where there is an effective HCV action plan, is the current lack of political will and dedicated funding to support expanded efforts to enhance HCV prevention and care for Canadians infected with HCV. It was universally agreed that a National Action Plan should be a focus for HCV priorities in Canada. Along these lines, it was highlighted that delivering HCV treatment in Canada involves dealing with 14 different government organizations that pay for HCV drugs, and with this in mind it was suggested that the PHAC would need to take a leadership role in developing a National strategic policy to guide HCV management in Canada. It was generally agreed that effective treatment dissemination will require the concerted efforts of public health and government policy makers, clinicians, nurses, advocacy groups, community groups, and civil society.

### 3.4. Action Hepatitis Canada: The Role of Patient Advocacy in Promoting HCV Diagnosis and Treatment

Daryl Luster (Steering/Executive Committee—Action Hepatitis Canada; President, Pacific HepC Network, Vancouver, BC, Canada) presented on the role of community advocacy in building a Canadian HCV action plan [46]. The take-home message of this presentation was that “person-centred healthcare” is key to meeting the needs of those affected, and, for this to occur, their voices need to be heard and respected. He reminded the symposium attendees that many of the affected communities are vulnerable and could benefit from assistance in expressing their needs and help navigating the system. Affected communities can learn from others who have successfully advocated for their cause; the HIV community provides an excellent example. Cross-sectional collaborations are considered to be essential, and Luster cited the Action Hepatitis Canada/Action Hépatite Canada (http://www.actionhepatitiscanada.ca/) as an example of how an umbrella group of organizations can coalesce so their common voices can be heard.

### 3.5. HCV Care and Treatment: The Role of the Hepatology Nurse

Magdalena Kuczynski (Toronto Western Hospital, Toronto, Canada) presented the hepatology nurse's viewpoint, outlining how nursing services need to support a person for more than just their treatment course [47]. Given the cost of treatment, completion of insurance or publically funded drug benefits approval forms, counselling patients about side effects (especially when IFN-based treatments are used), and ensuring that potential drug interactions are considered and addressed are just small part of the support patients need. Addressing these concerns is critical to maximize the ability of patients to be cured of HCV infection. Although IFN-free regimens are expected to herald an era of well-tolerated, short course treatments, many of the affected patients remain vulnerable. Patients and their families require educational support, help navigating the health system so they remain adherent, assistance in managing potential side effects, and continuity of care—including help to manage comorbid health conditions. Although the substantially improved side effect profile of IFN-free regimens is expected to decrease the need for nursing support from a treatment perspective, this is not the full story. The large population of aging baby boomers, many of whom have underlying comorbid illnesses, and treating people who inject drugs will require a broad range of harm reduction supports, including addictions and mental health services. As a result, strong nursing support will continue to be required to link clinical care with the social and the psychological supports required to efficiently and effectively manage and cure those who are HCV-infected.

### 3.6. Social and Behavioural Sciences: HCV Treatment and Diagnosis in Vulnerable Populations

The majority of new and existing cases of HCV in Canada occur among current or former PWID [48, 49]. Furthermore, although simple, tolerable, and short duration IFN-free HCV therapies have demonstrated high efficacy (>90%) in clinical trials [25], the effectiveness of IFN-based therapies in “real-world” settings is historically lower than the efficacy reported from clinical trials [50]. Attempts to understand why HCV transmission continues to occur among PWID and why clinical trial efficacy data does not play out in “real-world” settings require an understanding of the social and behavioural influences that put people at risk of infection or lead to nonadherence to therapy.

Medical anthropologist Dr. Philippe Bourgois (University of Pennsylvania, Philadelphia, USA) explored how cultural relativism, structural vulnerability, and risk environment shape individual behaviours among PWID [51]. Drawing on concepts from medical anthropology and using participant observation methodologies, Dr. Bourgois followed a social network of PWID from 1994 to 2007, analysing their survival strategies and their interactions with medical services and law enforcement. He highlighted the importance of the concept of cultural relativism (e.g., the fact that no culture is good or bad, but all have logic), the need to recognize the logic of competing/contradictory priorities that lead to unhealthy outcomes, and the structural forces shaping individual behaviours. He also emphasized the importance of structural vulnerability and risk environment, which are forces that render patients subject to negative health and limit personal ability/agency to make positive choices (from medicine's point of view). Drawing from his field work, he illustrated how law enforcement can present particular issues in terms of increasing structural vulnerability and the risk environment for people acquiring HCV infection. However, given the high prevalence and incidence of HCV infection in prisons [52], he also suggested that the prison setting paradoxically offers a unique opportunity for HCV treatment, particularly with respect to using the “treatment as prevention” approach. But, given the high cost of IFN-free regimens, considerable advocacy will be required before HCV treatment as prevention can be considered on a broader scale [53].

Any attempt to scale up HCV treatment will require strategies targeting enhanced testing, linkage to care, and assessment of HCV-related liver disease, which has been long complicated by the fact that liver biopsy is invasive and logistically difficult. However, the availability of noninvasive fibrosis assessment methods such as transient elastography (e.g., Fibroscan®) has greatly improved the ease of liver disease assessment. Transient elastography has excellent utility for the identification of HCV-related cirrhosis [54], can predict HCV-related survival [55], and is cost-effective [56]. In a presentation by Dr. Grebely (University of New South Wales, Sydney, Australia), the feasibility of noninvasive liver disease assessment (Fibroscan) among people with a history of injecting drug use participating in a liver health promotion campaign at three drug and alcohol clinics and a medically supervised injecting centre was evaluated [57]. Overall, among 252 people assessed for liver disease, 20% demonstrated advanced liver disease (stage F3/F4) and 60% returned for clinical follow-up within three months following assessment. This is consistent with several studies demonstrating that transient elastography is a useful tool for enhancing liver disease screening among PWID attending drug and alcohol clinics [58, 59]. Collectively, these data suggest that increased community-based liver disease screening using transient elastography might be one useful strategy for linking PWID into HCV care and triaging those with advanced liver disease who are in need of immediate treatment.

### 3.7. Epidemiology and Public Health: Strategies to Reduce HCV Burden in Canada

In the era of IFN-free DAA therapies, elimination of HCV is now a possibility. Dr. Rob Myers (University of Calgary, Calgary, Alberta, Canada, and presently Senior Director at Gilead Sciences, San Francisco, CA, USA) presented the landscape of the Canadian HCV burden and proposed management strategies that could eliminate HCV by 2035 [60]. As of 2013, taking into account high-risk populations (incarcerated individuals, Aboriginals, and PWID), it is estimated that the prevalence of chronic HCV is ~0.8% of the Canadian population. Due to the aging population, the clinical burden of liver disease is increasing. Modeling suggests that over the next two decades, cases of decompensated cirrhosis, hepatocellular carcinoma, and liver-related mortality will increase drastically if current treatment uptake is continued (~3600 treatments/year) [41]. Fortunately, as we transition to simpler and more tolerable all oral therapies to treat HCV, there is a possibility of changing this landscape. Dr. Myers evaluated different treatment uptake scenarios and concluded that increasing treatment uptake to >10,800 patients per year (3 times current practice) over the next 20 years could eliminate HCV in Canada. However, the main limitation will be identifying patients to treat. This will require increasing the capacity of diagnosis, treatment, and funding. Hence, this is an enormous undertaking and without reliable population-level cost data on liver disease complications it will be difficult to estimate cost effectiveness.

Lessons can be learned from bold public health initiatives such as the Scottish Hepatitis C Action Plan. Professor Sharon Hutchinson (Glasgow Caledonian University, Scotland, UK) illustrated how, after only 6 years, a strategic and well-executed action plan with the full support of the government and adequate funding (around £100 million) can transform services and rapidly improve the lives of thousands [61, 62]. The plan focused on PWID (including those continuing to inject and those who have ceased injecting), the population at highest risk of transmission, which represents 90% of the Scottish chronic hepatitis C population. The plan was divided into three phases with three main public health objectives in mind: prevention, diagnosis, and treatment [62]. Initiatives ranged from increasing and improving the provision of injecting equipment (needles/syringes and other paraphernalia used to prepare drugs for infection) and introducing finger-prick blood sampling by nonclinical staff in community settings to establishing targets to ensure rapid scale-up of antiviral therapy. Clear and informative indicators were used to monitor the plan, describing the numbers infected, diagnosed, and treated but also included more penetrative data on end-stage liver disease and death. Models were developed using this data to demonstrate the beneficial impact of scaling-up therapy on serious outcome trajectories. Achievements included an approximately 50% increase in the proportion of the infected population diagnosed (38% to 55%); a sustained 2.5-fold increase in the annual number of people initiating HCV therapy (450 to 1100), with more pronounced increases among PWID (300 to 900) and incarcerated individuals (20 to 140); and a reduction in the overall number of people living with chronic infection (39,000 to 37,000) [62]. Although the 3% treatment coverage is still relatively low, this is still more than twice the current rate in Canada (~1.4%). As in most countries, Scotland still faces numerous barriers: many individuals are still not aware of their diagnosis (primarily among people aged 35–64 years); there is a shortfall in patients reaching and staying in specialist care; and the current cost of treatment is prohibitive. However, their impressive achievements illustrate the power of a united and well-funded government supported program.

### 3.8. Funding for HCV Research in Canada

Dr. Marc Ouellette (Scientific Director, CIHR Institute of Infection and Immunity) addressed the contributions of the CIHR to HCV research in Canada [63]. The CIHR has provided over $64 million over the last 5 years for HCV research [63], primarily to fund projects focused on biomedical and clinical research questions. New funding initiatives in the field will aim to provide balance across CIHR's four health research pillars. CIHR's long-standing partnership with PHAC has supported a broad spectrum of hepatitis C-related research ($16 million through the partnership since 1999), establishing policies and priorities for supporting HCV research, expanding Canadian research capacity and productivity through training support, and encouraging the dissemination and uptake of research results. In December 2014, CIHR and PHAC announced their intention to build on the success of previous investments made through their partnership, including the NCRTP-HepC program, and support the creation and operation of a National Hepatitis C Research Network. The goal of this new initiative is to develop a cohesive, collaborative research program in Canada that links researchers, knowledge users, and decision makers from multiple pillars to facilitate integrated knowledge creation and translation approaches, while improving the overall environment for HCV research in Canada. In July 2015, the National Hepatitis C Collaborative Network (NHCCN) was funded through this initiative. The NHCCN will continue to lead the NCRTP-HepC, organize the annual Canadian Symposium on hepatitis C virus, and will also coordinate collaborative and transdisciplinary HCV research projects.

### 3.9. Update on the Proposed National HCV Task Group

Dr. Mel Krajden (University of British Columbia, BC, Canada) reported on the status of the Proposed National HCV Task Group whose intended role was to create a National HCV response in Canada [64]. It is important to note that both the US Centers for Disease Control (CDC) in Atlanta in 2012 and the US Preventative Services Task Force endorsed a one-time HCV baby boomer (1945–1965) screening recommendation [65]. This endorsement was based on evidence that a significant number of baby boomers are HCV infected and unaware of their status and that these individuals would benefit from curative treatment. At the 2nd CSHCV in Victoria [2], Canadian HCV surveillance data was presented by PHAC who then supported a meeting to update the Canadian HCV burden of disease data (Oct 2013). Although a Canadian 1945–1970 age cohort one-time screening recommendation was due to be released by PHAC on Dec 18, 2014, the recommendation was held back due to a lack of consensus amongst the provinces and territories on the benefits and how best to implement one-time screening [3]. A challenge with the Canadian single payer system is that decisions which may reflect the ethical “duty to inform” people about new standards of care is often juxtaposed against the societal and payer perspective that tries to balance boundless healthcare demands against finite resources.

The Council of Chief Medical Officers of Health initially recommended the creation of a National HCV Task Group (July 2014) and this was discussed by the Pan-Canadian Public Health Network. However, there were concerns expressed by many provinces that the work of a National HCV Task Group would overlap with activities of other working groups, such as the Canadian Preventative Health Task Force and provinces carrying out their own HCV treatment and cost-effectiveness assessments. As a result, a National HCV Task Group was not convened. Unresolved national-level questions surrounding HCV include

- reviewing Canadian HCV epidemiology to account for provincial/territorial and affected population disease burden differences;
- determining how best to implement one-time screening on top of the current risk-based testing;
- estimating the incremental yield of one-time testing relative to prioritizing the treatment of those already diagnosed;
- assessing whether “treatment as prevention” might reduce onward transmission amongst people who inject drugs;
- determining surveillance and healthcare provider capacity needs;
- providing phased options for increased treatment uptake based on Canadian cost/benefit analyses.

This information could provide guidance to the Council of Deputy Ministers who are the key decision makers. Alas, Canada's current response to HCV more closely resembles ten provinces and three territories acting as their own country, rather than a Nation committed to a unified solution.

## 4. Outcomes of the 4th CSHCV

Even a few years ago, the development and approval of IFN-free HCV therapies was a distant goal. Today, with combinations of DAAs, we can now cure most, if not all, individuals infected with HCV. However, these therapies are extremely costly and without a reduction in price it will not be possible to treat the entire Canadian HCV-infected population. Current challenges are focused on how to implement these treatments in a way that best benefits affected Canadians and Canadian society. Public funding for optimal DAA combinations varies across the country, with negotiations occurring independently in every province. All provinces and territories have now developed policies to fund treatment with DAA combinations for HCV patients with liver fibrosis (with a severity of F2 or above). However, this is in stark contrast to other settings such as Australia, where the Pharmaceutical Benefits Advisory Committee has recommended the approval of combinations of IFN-free therapy without liver disease-based restrictions. Patient advocacy groups will be instrumental in driving policies on who will receive treatment. In addition, the increased demand for treatment and the underlying comorbidities in the Canadian HCV-infected population will impose a high burden on healthcare practitioners.

The fight against HCV is not a one-size-fits-all approach. Challenges still remain in developing strategies to engage and treat members of marginalized populations in whom rates of HCV infection are the highest, including PWID and incarcerated individuals. Strategies will also be required to engage indigenous populations in treatment and care in a manner that respects their unique traditions and needs. It is time for Canada to develop a comprehensive and effective program tailored to the scale and needs of the population at risk. A national strategy would streamline the adoption of new HCV therapies and unify Canada in creating policies for their use.

## Figures and Tables

**Table 1:** Sessions, topics, and speakers for the 4th Canadian Symposium on hepatitis C virus discussed in this report.

Session	Topic	Speaker	Institution
Clinical Sciences	HCV Treatment in the Era of Highly Effective Antiviral Therapy	Mark Sulkowski	Johns Hopkins University (Baltimore, USA)
	HCV Care Clarity and Chaos in Canada	Curtis Cooper	University of Ottawa (Ottawa, Canada)
	Efficacy of Sofosbuvir Treatment Regimens in Real Life Settings	Emmanuelle Huchet	Clinique l'Actuel (Montréal, Canada)

Biomedical Sciences	Viral and Host Factors of Hepatitis C Virus RNA Replication	Volker Lohmann	University of Heidelberg (Heidelberg, Germany)
	Resistance to HCV NS5A and NS5B Inhibitors	Matthias Götte	University of Alberta (Edmonton, Canada)

Behavioural Sciences	Contradictions between Law Enforcement and Public Health: The Hepatitis C Risk Environment	Philippe Bourgois	University of Pennsylvania (Philadelphia, USA)
	LiveRLife: A Liver Health Promotion Campaign	Jason Grebely	University of New South Wales (Sydney, Australia)

Epidemiology and Public Health	Scotland's Action Plan on Hepatitis C	Sharon Hutchinson	Glasgow Caledonian University (Glasgow, Scotland)
	Burden of HCV in Canada and Management Strategies	Rob Myers	University of Calgary (Calgary, Canada)

A Plan for Canada	Beyond the Medication: Resources Needed for Successful Treatment	Magdalena Kuczynski	Toronto Western Hospital (Toronto, Canada)
	HCV Patient Advocacy in Canada	Daryl Luster	Action Hepatitis Canada (Vancouver, Canada)
	Status of the National HCV Task Group	Mel Krajden	University of British Columbia (Vancouver, Canada)
	CIHR Funding for HCV Research in Canada	Marc Ouellette	Canadian Institutes of Health Research (CIHR) (Québec, Canada)

Videos of the presentations are available at https://www.youtube.com/channel/UCUgCySYhpXIUuqiaQS_rGJw.
